# Serum gamma-glutamyltransferase is not associated with subclinical atherosclerosis in patients with type 2 diabetes

**DOI:** 10.1186/s12933-016-0426-1

**Published:** 2016-08-05

**Authors:** Hye Eun Yoon, Eun Young Mo, Seok Joon Shin, Sung Dae Moon, Je Ho Han, Eun Sook Kim

**Affiliations:** 1 Department of Internal Medicine, College of Medicine, The Catholic University of Korea, Seoul, Republic of Korea; 2 Division of Nephrology, Department of Internal Medicine, Incheon St. Mary’s Hospital, Incheon, Republic of Korea; 3 Division of Endocrinology and Metabolism, Incheon St. Mary’s Hospital, College of Medicine, The Catholic University of Korea, 222 Banpo-daero, Seocho-gu, Seoul, 137-071 Seoul Republic of Korea

## Abstract

**Background:**

This study investigated the association between serum gamma-glutamyltransferase (GGT) level and subclinical atherosclerosis in patients with type 2 diabetes.

**Methods:**

This cross-sectional study involved 1024 patients with type 2 diabetes mellitus. Measurement of brachial-ankle pulse wave velocity (baPWV; as a marker of arterial stiffness) and an ultrasound assessment of carotid atherosclerosis were performed. Subclinical atherosclerosis was defined by the presence of a high baPWV (≥1720 cm/s), carotid atherosclerosis (intima-media thickness >0.8 mm or the presence of plaques), and carotid stenosis (≥50 % of luminal narrowing). The subjects were stratified into quartiles according to GGT level, and the relationship between GGT level and subclinical atherosclerosis was analysed.

**Results:**

Serum GGT levels were closely associated with obesity, atherogenic dyslipidemia, and metabolic syndrome. However, serum GGT levels did not show a linear association with baPWV, carotid intima-media thickness, or plaque grade. The prevalence of high baPWV, carotid atherosclerosis, and carotid stenosis did not differ between the quartiles in men and women. Multivariate logistic regression analyses revealed no association between GGT level and high baPWV, carotid atherosclerosis, and carotid stenosis, either as continuous variables or quartiles.

**Conclusions:**

Serum GGT levels were significantly associated with obesity, atherogenic dyslipidaemia, and metabolic syndrome, but not with the early and late stages of atherosclerotic vascular changes, in patients with type 2 diabetes. Serum GGT level may not be a reliable marker of subclinical atherosclerosis in type 2 diabetes.

## Background

Gamma-glutamyltransferase (GGT) is a membrane-bound enzyme that plays a primary role in the regeneration of intracellular glutathione (GSH), a major cellular antioxidant [1–3]. Serum GGT level is a well-known marker of liver disease and excessive alcohol consumption. Recently, growing evidence has indicated that GGT level is closely associated with cardiovascular disease (CVD) in a dose-dependent manner, and even suggests a predictive role regarding mortality independent of conventional risk factors, both in general and specific populations [4–8]. A meta-analysis of seven prospective observational studies including 273,141 participants reported that GGT was associated with increased cardiovascular (CV) mortality (adjust relative risk of 1.52 for CV mortality and 1.56 for all-cause mortality in the highest vs lowest quartile of GGT) [9].

Patients with type 2 diabetes mellitus (T2DM) suffer from a 2–4 times greater burden of CVD compared with those without diabetes [10]. Thus, guidelines recommend a multifactorial intervention targeting glucose, lipid profiles, blood pressure levels, and established CVD risk factors in treating patients with T2DM. However, as the conventional risk factors do not fully explain the excess risk of mortality observed in T2DM, there is an increasing demand for identifying additional markers, for better CV risk assessment [11]. In this light, it is important to determine whether GGT level is an independent marker of atherosclerotic vascular changes in T2DM patients.

Increased carotid intima-media thickness (IMT) and arterial stiffness are both surrogate markers of changes in vascular morphology and function [12]. Carotid ultrasonography and measurement of brachial-ankle pulse wave velocity (baPWV) are simple, non-invasive modalities that are used to measure carotid IMT and arterial stiffness, respectively, with good predictive power for cardiovascular outcome; they are also widely accepted as efficient tools for identifying subjects at a high risk for CVD in clinical practice [13, 14]. A few epidemiological studies examined the relationship between serum GGT level and arterial stiffness, but the findings were inconsistent in terms of sex-specific associations [15–19]. Previous studies on the association between serum GGT level and carotid IMT yielded inconsistent results [20–23]. However, to our knowledge, no study has assessed simultaneously different parameters of subclinical atherosclerosis to demonstrate the relationship between serum GGT level and subclinical atherosclerosis in patients with TD2M, who have an increased risk of CVD [24].

In this study, we investigated the associations between serum GGT level and vascular wall properties in patients with T2DM by measuring baPWV and performing an ultrasound assessment of carotid atherosclerosis.

## Methods

### Subjects

We retrospectively recruited subjects with T2DM older than 30 years who visited Incheon St Mary’s Hospital for the purpose of glucose control between August 2011 and November 2013. Diabetes was diagnosed by the American Diabetes Association criteria [25]. A total of 1965 patients with measurements of GGT and subclinical atherosclerosis were enrolled in this study. The patients who were excluded were those with GGT levels >100 U/L (n = 98), an ankle-brachial index <0.9 or >1.3 (n = 48), elevated liver enzymes [aspartate aminotransferase (AST) >100 IU/L or alanine aminotransferase (ALT) >100 IU/L, n = 56], a history of cardiovascular, cerebrovascular, or peripheral artery disease (n = 235), chronic liver disease (n = 59), malignancy or taking warfarin or corticosteroids (n = 30), and those without waist circumference (WC) measurement (n = 415) were excluded. A total of 1024 patients were included in the final analysis. The study protocol was approved by the institutional review board of Incheon St. Mary’s Hospital (IRB No. OC15RISI0097).

### Clinical and biochemical assessment

Baseline demographic and clinical data, including age, sex, height, weight, co-morbidities, laboratory results, and therapeutic characteristics, were recorded. Body mass index (BMI) was calculated by dividing the patients’ weight in kilograms by their height in meters squared. WC was measured to the nearest 0.1 cm midway between the iliac crest and the lower rib margin using a non-stretchable tape, with participants standing erect. After overnight fasting, venous blood was taken, and blood analyses were processed within 2 h of blood collection. Serum GGT levels were analysed using an automated chemistry analyzer (AU5400, Beckman Coulter, Fullerton, USA). Fasting plasma glucose (FPG), haemoglobin A1c (HbA1c), AST, ALT, uric acid, total cholesterol (TC), triglyceride (TG), and high-density lipoprotein-cholesterol (HDL-C) were determined from blood samples. Low-density lipoprotein-cholesterol (LDL-C) was indirectly measured using the Friedewald formula only in participants with serum TG concentrations <400 mg/mL. Serum insulin was measured using a Roche Cobas E601 (Roche) instrument, and homeostasis model assessment of insulin resistance (HOMA-IR) was calculated as follows: HOMA-IR = fasting insulin (μU/mL) × FPG (mmol/L)/22.5. Hyperlipidemia was defined as a TG concentration of 150 mg/dL or greater or an LDL-C concentration of 100 mg/dL or greater and/or taking cholesterol-lowering medication. Patients were considered to have hypertension if they had a systolic blood pressure (SBP) of 140 mmHg or greater and/or a diastolic blood pressure (DBP) of 90 mm Hg or greater or if they were on treatment for the condition. We used the National Cholesterol Education Program-Adult Treatment Panel III criteria to determine the presence of metabolic syndrome (MetS) using the cut-offs for the Asia–Pacific region [26]. As all subjects had diabetes; MetS was considered to be present if two or more of the following conditions were present: (1) SBP/DBP ≥130/85 mmHg or on antihypertensive drug treatment; (2) fasting serum TG ≥150 mg/dL; (3) low HDL-C (<40 mg/dL in men and 50 mg/dL in women); and (4) WC ≥90 cm in men and ≥80 cm in women.

The estimated glomerular filtration rate (eGFR) was calculated based on the modification of diet in renal disease study equation [27].

### Measurement of baPWV

The baPWV was measured using an automated pulse wave velocity (PWV)/ankle-brachial index analyzer (VP-2000; Colin Co Ltd) after the subjects had rested in the supine position for at least 5 min. The electrocardiogram electrodes were placed on both wrists and both ankles, and blood pressure cuffs were wrapped around both upper arms and both ankles. To measure the baPWV, pulse waves obtained from the brachial and tibial arteries were recorded simultaneously, and the transmission time was calculated as the time interval between the initial increase in the brachial and ankle waveforms. The path length from the suprasternal notch to the brachium and from the suprasternal notch to the ankle was automatically obtained based on the subjects’ height. The baPWV was calculated using the equation baPWV = (length from the suprasternal notch to the ankle − length from the suprasternal notch to the brachium)/transmission time (cm/s), and the mean baPWVs for the left and right sides were used for the analysis. A high baPWV was defined as the highest quartile of values among the subjects (≥1720 cm/s).

### Ultrasonic assessment of carotid artery disease

Carotid IMT was measured bilaterally using a high-resolution B-mode scanner (ALOKA; ProSound Alpha 10) with a 10 MHz transducer. Carotid IMT measurement and plaque assessment were performed as recommended by the Manheim Carotid Intima–Media Thickness and the American Society of Echocardiography Consensus. IMT was measured from three contiguous sites at the region 10–20 mm proximal to the carotid bulb, and the mean IMT was calculated as the average of right and left mean IMT. Plaque was defined as a localized or broad lesion, and the broad lesion was defined as >50 % of the surrounding IMT or a thickness of 1.5 mm. The method suggested by Chien et al. [28] was used for plaque quantification scoring. In brief, common carotid artery (CCA) segments, including the proximal CCA, distal CCA, bulb, internal carotid artery, and external carotid artery, were examined bilaterally. A grade was assigned for each segment: grade 0 for normal or no observable plaque; grade 1 for one small plaque with diameter stenosis <30 %; grade 2 for one medium plaque with 30–49 % diameter stenosis or multiple small plaques; grade 3 for one large plaque with 50–99 % diameter stenosis or multiple plaques with at least one medium plaque; and grade 4 for 100 % occlusion. The highest value at any segment was used for the analysis. We defined carotid atherosclerosis as a carotid IMT >0.8 mm or the presence of plaques. Carotid stenosis was defined as a luminal narrowing of 50 % or greater (plaque score >2).

### Statistical analyses

Serum GGT levels were classified into quartiles: ≤16, 17–23, 24–37, and ≥38 U/L. Differences in the baseline characteristics between the lowest GGT quartile and other quartiles were evaluated. Continuous data were expressed as the mean ± SD or as the median with interquartile range (25th–75th percentile) in case of skewed distribution, and were compared using one-way ANOVA or the Kruskal–Wallis test, as appropriate. Categorical data were expressed as numbers (percentage) and compared using the Chi squared test. Multivariate logistic regression analyses were performed to estimate the odds ratios (ORs) and 95 % confidence intervals (CIs) for high baPWV, carotid atherosclerosis, and carotid stenosis according to the GGT quartiles and an increase of 1 U in the GGT. Linear regression analysis was used to identify the independent predictors of baPWV, carotid IMT, and plaque grade. Age-adjusted means and 95 % CIs of GGT levels were calculated using ANCOVA according to each component of MetS, MetS, and number of MetS components that were met by the subjects. Logistic regression analysis was performed to evaluate the association between serum GGT level and MetS.

All statistical analyses were performed using SAS. A *P* < 0.05 was considered statistically significant.

## Results

### Clinical characteristics of the subjects

Table 1 shows the clinical characteristics of the study subjects according to the GGT quartile groups. Patients in the highest quartile group were younger and more likely to be male, were current smokers and alcohol drinkers, had a shorter duration of diabetes, and were less likely to be insulin users. Metabolic profiles, including BMI, WC, and levels of TC, TG, LDL-C, and uric acid, increased as the GGT quartiles increased. The glycaemic status measured by FPG and HbA1c increased across the GGT quartile groups. Subjects in the highest quartile group had higher levels of AST and ALT and higher eGFR and DBP. Regarding subclinical atherosclerosis, there were no significant differences in baPWV, carotid IMT, and carotid plaque grades across the quartiles.

**Table 1:** Characteristics of the study subjects

	GGT (U/L)				*P*
	Q1	Q2	Q3	Q4	
	≤16	17–23	24–37	≥38	
n	257	267	250	250	
Age (years)	58.6 ± 10.1	57.5 ± 10.0	55.6 ± 9.5	55.1 ± 10.0	<0.001
Male gender (%)	59 (23.0)	109 (40.8)	125 (50.0)	139 (55.6)	<0.001
Current smokers (%)	25 (9.7)	37 (13.9)	47 (18.8)	49 (19.6)	0.006
Alcohol (%)	47 (18.3)	87 (32.6)	112 (44.8)	122 (48.8)	<0.001
Duration (years)	9.4 (8.5–10.3)	8.1 (7.2–9.0)	6.7 (5.8–7.6)	6.0 (5.1–6.9)	<0.001
BMI (kg/m^2^)	23.9 ± 3.1	24.7 ± 3.4	25.6 ± 3.6	26.1 ± 3.6	<0.001
WC (cm)	83.5 ± 9.1	86.1 ± 8.8	88.7 ± 8.8	90.7 ± 9.6	<0.001
FPG (mg/dL)	146.3 ± 62.3	148.9 ± 56.6	161.5 ± 70.0	172.1 ± 68.9	<0.001
HbA1c (%)	7.9 ± 2.1	8.0 ± 2.2	8.1 ± 2.2	8.2 ± 2.0	0.345
HOMA-IR^a^	3.9 (2.6–5.2)	2.8 (1.6–4.1)	3.8 (2.6–5.1)	4.4 (3.2–5.6)	0.353
TC (mg/dL)	169.4 ± 44.6	173.1 ± 40.8	177.7 ± 43.7	185.6 ± 48.6	<0.001
TG (mg/dL)	127.1 (114.6–139.7)	148.8 (136.3–161.2)	170.3 (157.2–183.5)	199.0 (185.9–212.0)	<0.001
HDL-C (mg/dL)	48.1 ± 11.4	46.6 ± 12.1	46.0 ± 11.9	45.6 ± 11.5	0.111
LDL-C (mg/dL)	106.5 ± 33.4	105.2 ± 34.5	110.8 ± 32.7	117.3 ± 37.6	<0.001
Uric acid (mg/dL)	4.8 ± 1.3	4.9 ± 1.4	5.2 ± 1.5	5.3 ± 1.5	0.007
AST (U/L)	20.6 ± 5.7	22.6 ± 7.4	25.3 ± 9.6	31.1 ± 13.5	<0.001
ALT (U/L)	17.8 ± 8.0	22.9 ± 12.2	28.2 ± 14.3	36.3 ± 18.5	<0.001
eGFR (mL/min/1.73 m^2^)	101.8 ± 32.4	99.0 ± 28.2	101.2 ± 27.4	106.9 ± 29.5	0.021
SBP (mmHg)	129.6 ± 19.3	129.4 ± 17.0	129.1 ± 16.8	132.4 ± 16.9	0.264
DBP (mmHg)	76.5 ± 10.5	77.7 ± 10.1	78.7 ± 9.4	80.3 ± 10.3	<0.001
*Usage of medication (%)*					
Insulin	72 (28.0)	59 (22.1)	53 (21.2)	38 (17.1)	0.006
Anti-platelet agent	101 (39.3)	100 (37.5)	83 (33.2)	80 (32.0)	0.264
CCB	53 (20.6)	59 (22.1)	59 (23.6)	46 (18.4)	0.529
ACEI/ARB	93 (36.2)	84 (61.5)	95 (38.0)	85 (34.0)	0.436
Beta blocker	23 (9.0)	19 (7.1)	22 (8.8)	20 (8.0)	0.864
Statin	92 (35.8)	104 (39.0)	117 (46.8)	89 (35.6)	0.034
baPWV (cm/s)	1583 ± 338	1578 ± 329	1518 ± 322	1556 ± 323	0.102
IMT (mm)	0.65 ± 0.14	0.65 ± 0.14	0.67 ± 0.15	0.66 ± 0.14	0.663
*Carotid plaque (%)*					0.984
Grade 0	161 (62.7)	168 (62.9)	156 (62.4)	150 (60.0)	
Grade 1	23 (9.0)	22 (8.2)	16 (6.4)	22 (8.8)	
Grade 2	46 (17.9)	46 (17.2)	44 (17.6)	51 (20.4)	
Grade 3	25 (9.7)	29 (10.9)	32 (12.8)	26 (10.4)	
Grade 4	2 (0.8)	2 (0.8)	2 (0.8)	1 (0.4)	

Data are means ± SD or numbers (percentage)

*BMI* body mass index, *WC* waist circumference, *FPG* fasting plasma glucose, *HbA1c* hemoglobin A1c, *HOMA-IR* homeostasis model assessment of insulin resistance, *TC* total cholesterol, *TG* triglyceride, *HDL-C* high-density lipoprotein-cholesterol, *LDL-C* low-density lipoprotein-cholesterol, *AST* aspartate aminotransferase, *ALT* alanine transferase, *ALP* alkaline phosphatase, *SBP* systolic blood pressure, *DBP* diastolic blood pressure, *eGFR* estimated glomerular filtration rate, *CCB* calcium channel blocker, *ACEI/ARB* angiotensin-converting enzyme inhibitor/ angiotensin II receptor blocker, *CVD* cardiovascular disease, *FRS* Framingham Risk Score, *baPWV* brachial-ankle pulse wave velocity, *IMT* intima-media thickness

^a^N = 505

### Association between GGT level and MetS

Table 2 shows the age-adjusted means and 95 % CI of GGT levels according to each component of MetS, MetS, and number of MetS components. The GGT levels were significantly higher in subjects with increased WC and high TG and in subjects with MetS, both in men and women. The GGT levels increased significantly as the number of MetS components increased, both in men and women.

**Table 2:** Age-adjusted relationship of GGT levels with each component of MetS (elevated BP, increased WC, high TG, and low HDL-C), MetS, and number of MetS components

	Men	*P*	Women	*P*
	mean (95 % CI)		mean (95 % CI)	
*Elevated BP*				
No	31.8 (28.7–34.8)	0.157	27.0 (24.5–29.6)	0.424
Yes	34.5 (32.3–36.6)		25.8 (24.1–27.5)	
*Increased WC*				
No	31.5 (29.2–33.8)	0.007	21.7 (18.9–24.6)	<0.001
Yes	36.4 (33.7–39.1)		27.6 (26.0–29.2)	
*High TG*				
No	29.7 (27.4–32.0)	<0.001	23.4 (21.7–25.2)	<0.001
Yes	38.6 (35.9–41.1)		30.7 (28.5–33.0)	
*Low HDL-C*				
No	34.8 (32.5–37.0)	0.099	24.6 (22.4–26.9)	0.086
Yes	31.7 (28.9–34.5)		27.2 (25.4–29.0)	
*MetS*				
No	30.1 (27.2–32.9)	0.002	21.8 (18.6–25.1)	0.003
Yes	35.7 (33.5–37.9)		27.2 (25.6–28.8)	
*MetS No*				
0	29.1 (24.2–34.0)	0.035	17.6 (10.0–25.2)	0.001
1	30.6 (27.1–34.1)		22.7 (19.2–26.3)	
2	34.8 (31.5–38.1)		24.8 (22.2–27.3)	
3	36.0 (32.4–39.5)		27.5 (25.2–29.8)	
4	37.8 (32.1–43.5)		31.4 (27.7–35.0)	

*BP* blood pressure, *WC* waist circumference, *TG* triglyceride, *LDL-C* low-density lipoprotein-cholesterol, *MetS* metabolic syndrome, *Met No* number of metabolic syndrome components which the subjects satisfy except diabetes

**Fig. 1:**
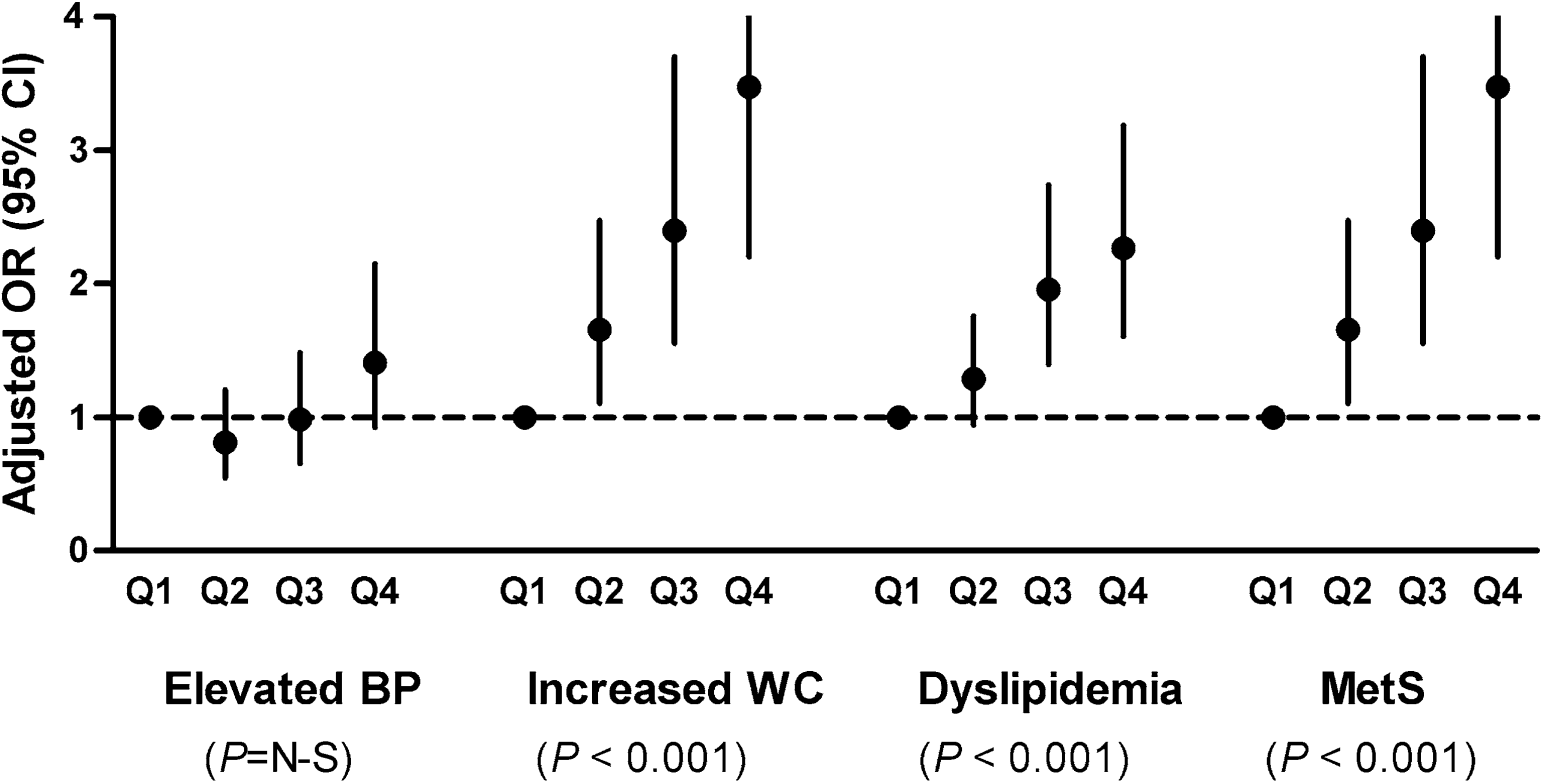
Logistic regression to evaluate the OR and 95 % CI for elevated BP, increased WC, atherogenic dyslipidemia, and MetS in GGT quartiles (*Q2*–*Q4*) compared to GGT *Q1*. As the GGT quartiles increased, the OR for increased WC, atherogenic dyslipidemia (high TG or low HDL-C), and MetS significantly increased. The multivariate model was adjusted for age, sex, smoking, alcohol consumption, diabetic duration, HbA1c, and eGFR

Figure 1 shows the OR and 95 % CI for each component of MetS and MetS, in GGT quartiles. As the GGT quartiles increased, the OR for increased WC, atherogenic dyslipidemia (high TG or low HDL-C), and MetS significantly increased after adjustments for age, sex, smoking, alcohol consumption, diabetic duration, HbA1c, and eGFR.

### Association between GGT level and high baPWV, carotid atherosclerosis, and carotid stenosis

**Fig. 2:**
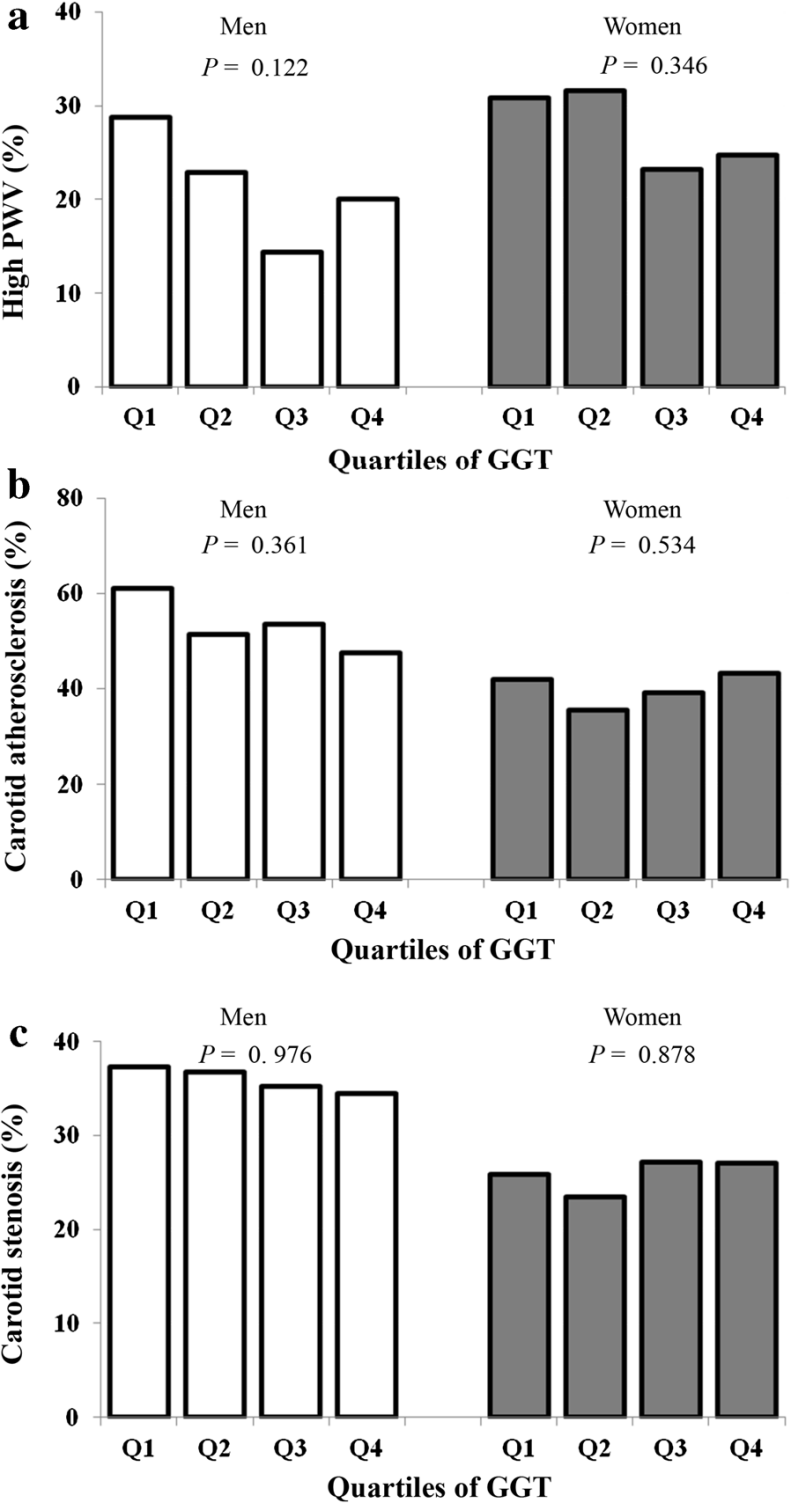
The prevalence of high baPWV (**a**), carotid atherosclerosis (**b**), and carotid stenosis (**c**) according to GGT quartiles in men and in women. The prevalence of high baPWV, carotid atherosclerosis, and carotid stenosis did not differ between the quartiles both in men and women

Figure 2 shows the prevalence of high baPWV, carotid atherosclerosis, and carotid stenosis according to GGT quartiles and sex. The prevalence of high baPWV, carotid atherosclerosis, and carotid stenosis did not differ between the quartiles, both in men and women.

Table 3 shows the OR and 95 % CI for high baPWV, carotid atherosclerosis, and carotid stenosis according to GGT levels, as continuous variables and quartiles. The risks of high baPWV, carotid atherosclerosis, and carotid stenosis were not correlated with GGT values, both as continuous variables and quartiles, in all models including cardiovascular risk factors, alcohol consumption, eGFR, and components of MetS.

**Table 3:** Odds ratios (95 % confidence intervals) for high baPWV, carotid atherosclerosis, and carotid stenosis according to quartiles of GGT

High baPWV^a^			Carotid atherosclerosis^b^		Carotid stenosis^c^	
OR (95 % CI)		*P*	OR (95 % CI)	*P*	OR (95 % CI)	*P*
*Non-adjusted*						
Per GGT (U)	1.01 (0.99–1.01)	0.718	1.00 (0.99–1.01)	0.824	1.00 (0.99–1.01)	0.581
Q1	1		1		1	
Q2	1.24 (0.85–1.81)	0.258	0.84 (0.59–1.18)	0.316	1.12 (0.65–1.93)	0.687
Q3	1.10 (0.75–1.62)	0.634	1.00 (0.71–1.42)	0.983	1.34 (0.78–2.30)	0.285
Q4	1.13 (0.75–1.69)	0.565	0.97 (0.69–1.38)	0.874	1.03 (0.59–1.81)	0.915
*Age and sex-adjusted*						
Per GGT (U)	1.00 (0.99–1.01)	0.980	1.00 (0.995–1.01)	0.584	1.00 (0.99–1.01)	0.793
Q1	1		1		1	
Q2	1.11 (0.71–1.71)	0.652	0.77 (0.53–1.13)	0.178	1.12 (0.64–1.98)	0.689
Q3	0.78 (0.49–1.26)	0.314	1.03 (0.70–1.52)	0.871	1.51 (0.85–2.67)	0.161
Q4	1.03 (0.65–1.64)	0.899	0.99 (0.67–1.47)	0.962	1.14 (0.63–2.08)	0.666
*Multivariate-adjusted*						
Per GGT (U)	1.00 (0.99–1.01)	0.988	1.00 (0.99–1.01)	0.911	1.00 (0.98–1.01)	0.394
Q1	1		1		1	
Q2	1.29 (0.80–2.08)	0.304	0.78 (0.53–1.16)	0.222	1.10 (0.62–1.97)	0.747
Q3	0.89 (0.53–1.52)	0.679	1.01 (0.67–1.53)	0.956	1.52 (0.78–2.58)	0.252
Q4	1.07 (0.63–1.82)	0.801	0.91 (0.60–1.39)	0.674	0.97 (0.51–1.84)	0.927

The multivariate model was adjusted for age, sex, smoking, alcohol consumption, use of statin, diabetic duration, HbA1c, eGFR, elevated blood pressure, increased waist circumference, high triglyceride levels, and low HDL-C

*baPWV* brachial-ankle pulse wave velocity

^a^Defined as the highest quartile of values among the subjects (≥1720 cm/s)

^b^Defined as cIMT > 0.8 mm or the presence of plaques

^c^Defined as 50 % or greater of luminal narrowing (plaque score ≥2)

Table 4 shows the multivariate linear regression analysis of baPWV, carotid IMT, and plaque grade. Serum GGT levels did not exhibit a linear association with baPWV, carotid IMT, or plaque grade, both in model 1 and 2.

**Table 4:** Linear regression analysis for baPWV, carotid IMT, or plaque grade

	baPWV		Carotid IMT		Plaque grade	
	*β* ^a^	*P*	*β*	*P*	*β*	*P*
*Model 1*						
Age	0.44	<0.001	0.46	<0.001	0.31	<0.001
Male gender	−0.05	0.119	0.23	<0.001	0.19	<0.001
Diabetic duration	0.13	<0.001	−0.01	0.727	0.03	0.416
HbA1c	0.05	0.045	0.07	0.015	0.04	0.214
Elevated BP	0.23	<0.001	0.07	0.009	0.09	0.003
Increased WC	−0.03	0.257	0.04	0.183	0.02	0.448
High TG	0.03	0.291	0.02	0.483	0.05	0.105
Low HDL-C	−0.02	0.376	0.05	0.059	−0.04	0.190
GGT	0.05	0.084	0.02	0.540	−0.003	0.923
R^2^	39.4		25.1		12.7	
*Model 2*						
Age	0.47	<0.001	0.46	<0.001	0.32	<0.001
Male gender	−0.01	0.756	0.23	<0.001	0.21	<0.001
Diabetic duration	0.15	<0.001	−0.01	0.805	0.03	0.326
HbA1c	0.04	0.096	*0.07*	0.011	0.04	0.255
MetS No.	*0.11*	<0.001	0.11	<0.001	0.07	0.027
GGT	0.04	0.154	0.01	0.657	0.003	0.935
R^2^	35.2		25.0		11.8	

*BP* blood pressure, *WC* waist circumference, *TG* triglyceride, *LDL-C* low-density lipoprotein-cholesterol, *HbA1c* hemoglobin A1c, *MetS* metabolic syndrome, *Met No* number of metabolic syndrome components which the subjects satisfy except diabetes, *β* ^*a*^ standardized coefficient, *β* unstandardized coefficient

*Model 1* The multivariate model included GGT, age, sex, smoking, alcohol consumption, use of statin, diabetic duration, HbA1c, eGFR, elevated blood pressure, increased waist circumference, high triglycerides, and low HDL-C

*Model 2* The multivariate model included GGT, age, sex, smoking, alcohol consumption, use of statin, diabetic duration, HbA1c, eGFR, and number of metabolic syndrome components (elevated blood pressure, increased waist circumference, high triglycerides and low HDL-C)

## Discussion

The results of this study indicate that serum GGT levels are significantly associated with obesity, atherogenic dyslipidaemia, and MetS in patients with T2DM. However, there was no significant association between GGT level and baPWV, carotid atherosclerosis, and carotid stenosis. BaPWV is a reliable measurement of arterial stiffness and has been proposed as an important mechanism for the diabetes-related increase in CV risk and mortality [29]. Increased IMT reflects an early stage of atherosclerosis related with intima hypertrophy or media-adaptive hypertrophy, and plaque formation reflects a late stage of atherosclerosis [30]. Thus, these findings demonstrate that GGT level is not a reliable marker of either early or late atherovascular changes in patients with T2DM, and underscores its doubtful role in the development of CVD in this population.

### GGT level and atherosclerosis

Recent studies highlighted the predictive role of serum GGT level in coronary heart disease, stroke, and increased mortality in both general and at-risk populations, regardless of sex, and suggest that GGT contributes to the process of atherosclerosis [2]. The possibility of an etiological association between GGT and mortality through CVD was supported by the positive association observed between serum GGT level and CVD risk factors such as diabetes, hypertension, dyslipidaemia, and MetS in epidemiologic studies [15–19]. The physiological characteristic of GGT also suggests its pathogenic role in the atherosclerotic process. GGT levels may be indirectly linked to atherosclerosis via coexistent oxidative stress, a well-known common mediator of vascular injury. An increased serum GGT level may be followed by cellular GGT overexpression to compensate for the exhausted glutathione, as a defence against elevated levels of reactive oxygen species. It has been reported that GGT is induced in response to elevated reactive oxygen species in tissues such as the liver and lung, and GGT level is correlated negatively with glutathione levels, as well as with either serum levels or consumption of antioxidant nutrients [31]. The predictability of serum GGT level for future concentration of F2-isoprostanes, a specific marker of oxidative damage, suggests that elevated GGT levels increase, rather than decrease, free radical formation as a net result [31]. Furthermore, recent studies indicate that GGT may participate directly in plaque progression and atherogenesis [2, 32]. Histological studies found that atherosclerotic plaques contain active GGT and co-localize with oxidized LDL and CD68^+^ foam cells [2], postulating that GGT-dependent pro-oxidant reactions may occur within the plaque and influence plaque progression and vulnerability, particularly in the presence of free iron [33].

### Previous studies on the associations between GGT level and arterial stiffness, carotid atherosclerosis, and carotid stenosis

Previous studies examined the association between GGT level and functional and morphological changes in the vasculature, mostly in the general population. It was reported that patients with increased coronary calcification levels were more likely to present with higher GGT values [34]. Recently a common GGT1 gene variant in T2DM subjects was shown to have significant effects on a high baPWV and diabetic retinopathy with interaction with a low HDL-C level [35]. Previous studies reported a positive association between GGT level and arterial stiffness, although there were differences in sex-specific associations [15–19], whereas inconsistent results were obtained regarding the association between GGT level and carotid IMT or plaque; some reports showed a significant association [20, 21], while others showed the opposite result [22, 23]. We could not provide the exact reasons for the lack of association observed between GGT level and subclinical atherosclerosis in this study; however, it might be primarily related to differences in the study population used. Our study subjects were a homogenous population with a diagnosis of T2DM. In contrast, previous reports included both diabetic and non-diabetic subjects, with the former being the minority (<11 %) [15, 17–20], or they excluded patients taking insulin-sensitizing medication or those with diabetes [16, 21].

### The clinical significance of serum GGT level in T2DM

Since moderate obesity was associated more strongly with a lower risk of mortality than with normal, underweight, and overweight groups in the general population of South Korea (so called “obesity paradox”) [36], we performed association analyses of serum GGT level for baPWV, carotid IMT, and plaque grade after subdividing patients according to the BMI levels (data not shown). However, there was no risk difference in GGT level between non-obese and obese subjects in this study. Considering the close association observed between GGT level and glucose intolerance [37], obesity [38], and MetS [39, 40], our findings suggest that serum GGT level reflects a metabolic status rather than an atherogenic one in the diabetic population. Subjects with T2DM have not only advanced subclinical atherosclerosis, such as high arterial stiffness, carotid IMT, or plaque, but also higher GGT levels compared with the non-diabetes population. Subjects with diabetes have been reported to have a two to fourfold increased risk of CVD [10], which is higher than the CV risk of GGT in the general population, as the pooled relative risk of CV mortality for GGT was 1.75 [9]. Thus, serum GGT levels may not add an additional formation on elevated CV risk in subjects who already have a high atherovascular burden. Regarding carotid IMT, Nuti et al. [23] have reported a lack of association with GGT level in 578 patients with hypertension and/or diabetes. Alternatively, serum GGT level, which comprises four fractions (b-, m-, s- and f-GGT), may not reflect GGT activity in atherosclerotic plaques. It has been reported that only b-GGT is found within atherosclerotic plaques and is associated with cardiovascular risk factors [41].

The interpretation of our data requires caution. Although there was a lack of association between serum GGT and atherosclerotic vascular changes, the present results do not mean that serum GGT level is not a marker of mortality in high-risk populations, either from CVD or all causes, in subjects with T2DM. A recent report showed that GGT is associated with incident CVD in people with T2DM [8], but controversial data exist [42]. Elevated GGT level was independently associated with the risk of 3-year all-cause mortality in patients with diabetes and coronary artery disease treated with percutanoues coronary intervention [43]. GGT level was also reported to be a risk factor for CV mortality in Japanese men and women independently from alcohol consumption [44]. Nevertheless, our results raise the possibility that the association between serum GGT level and the development of CVD may stem from an indirect link via metabolic profiles, rather than a direct involvement of GGT in atherogenesis. Further longitudinal and population-based studies could help determine whether GGT improves the predictability of CVD in the diabetic population.

## Limitations

This study had some limitations. First, it was a cross-sectional analysis, which cannot prove any causal relationship between GGT level and subclinical atherosclerosis. Second, only single GGT measurements were available, which is not as desirable as using the mean of several measurements. Third, although subjects with a history of liver disease were excluded from the study, non-alcoholic fatty liver disease detected by abdominal ultrasound was not fully evaluated. Despite these limitations, this study had some strengths: it was the first to evaluate the association between GGT level and subclinical atherosclerosis in T2DM, and simultaneous assessments of arterial stiffness, carotid atherosclerosis, and carotid stenosis were performed.

## Conclusion

Our results showed that serum GGT level was not associated with high baPWV, carotid IMT, or carotid plaque in patients with T2DM, suggesting that serum GGT level is not a reliable marker of subclinical atherosclerosis in T2DM. Further studies are needed to confirm whether GGT is a marker of future CVD in populations with diabetes.

## Abbreviations

GGT: gamma-glutamyltransferase
CVD: cardiovascular disease
CV: cardiovascular
T2DM: type 2 diabetes
IMT: carotid intima-media thickness
baPWV: brachial-ankle pulse wave velocity
AST: aspartate aminotransferase
ALT: alanine aminotransferase
WC: waist circumference
BMI: body mass index
FPG: fasting plasma glucose
HbA1c: haemoglobin A1c
TC: total cholesterol
TG: triglyceride
HDL-C: high-density lipoprotein-cholesterol
LDL-C: low-density lipoprotein-cholesterol
HOMA-IR: homeostasis model assessment of insulin resistance
SBP: systolic blood pressure
DBP: diastolic blood pressure
MetS: metabolic syndrome
eGFR: estimated glomerular filtration rate
PWV: pulse wave velocity
CCA: common carotid artery
OR: odds ratio
CI: confidence interval
ACEI: angiotensin-converting enzyme inhibitor
ARB: angiotensin II receptor blocker
Met No: number of metabolic syndrome components which the subjects satisfy except diabetes
